# Reviewed article: Campos HL, Fernandes GJA, Sousa DG, Conrado PLM, Campos EO, Santos Filho RAB, Costa PFF, Silva CM, Conrado GAM, Galvão PVM. Impacts of the COVID-19 pandemic on excess deaths: descriptive study, Pernambuco, 2020-2022. Epidemiol Serv Saude. 2025:34;e20240286

**DOI:** 10.1590/S2237-96222025v34e20240286.b

**Published:** 2025-09-01

**Authors:** Kaciane Corrêa Mochizuke

**Affiliations:** 1 Prefeitura Municipal de Porto Murtinho, Porto Murtinho, MS, Brazil

## Peer review B

## Questionnaire

Does the manuscript contain new and significant information to justify publication? No

Does the Abstract (Summary) clearly and accurately describe the content of the article? No

Is the problem significant and concisely stated? No

Are the methods described comprehensively? Yes

Are the interpretations and conclusions justified by the results? Yes

Is adequate reference made to other work in the field? Yes

## Manuscript Structure

Length of article is: Adequate 

Number of tables is: Adequate 

Number of figures is: Adequate

Please state any conflict(s) of interest that you have in relation to the review of this paper: None

**Interest:** Below Average

**Quality**: Below Average

**Originality:** Below Poor

**Overall:** Below Average

**Recommendation:** Reject

## Comments

O estudo foi classificado como ecológico, porém sua metodologia se assemelha a um estudo descritivo, que têm por objetivo determinar a distribuição de doenças ou condições relacionadas à saúde, segundo o tempo, o lugar e/ou as características dos indivíduos, o que vai ao encontro dos objetivos propostos do estudo em tela, devendo ser corrigido seu enquadramento para estudo descritivo.

Os objetivos do estudo são: ”estimar o excesso de óbitos durante a pandemia de COVID-19 no estado de Pernambuco, Brasil, entre os anos de 2020 e 2022”. Não deixa claro a escolha do estado para realização da estimativa uma vez que o excesso de óbitos foi observado globalmente. Sugiro que os autores revisem e considerem repensar a justificativa e o recorte do estudo para um objetivo capaz de agregar relevância, aplicabilidade e contribuições práticas ao SUS.

Descreva os métodos com maior rigor e detalhes, incluindo as variáveis analisadas, critérios de inclusão e exclusão e justifique suas escolhas, para além de trazer reprodutibilidade embasar a metodologia utilizada. Especifique e cite quais os CID’s analisados, esclareça o que o é considerado como motivo de óbito “ligado à COVID” de maneira clara.

Os métodos estatísticos são frágeis, a escolha da aplicação da média simples empobrece a metodologia. Procure lançar mão de testes estatísticos que demostrem adequadamente a probabilidade de se observar uma diferença significativa ou não.

As tabelas precisam ser revisadas para garantir que atendam aos requisitos da revista, vide ativos digitais - títulos de ilustrações.

A conclusão e o os descritores utilizados, bem como o número de palavras, número de tabelas e número de referências estão adequados aos requisitados pela revista.

O trabalho não apresenta novas perspectivas, conhecimentos ou descobertas relevantes para a área de estudo, não conferindo originalidade. Sugiro abordar novas perspectivas para a temática a fim de trazer relevância. Explique as implicações práticas das descobertas, discutindo sua relevância para a prática de medidas de saúde adequadas e como os resultados podem ser aplicados no contexto do SUS.

